# Engraftment syndrome following Hematopoietic stem cell transplantation: a systematic approach toward diagnosis and management

**DOI:** 10.1007/s12032-022-01894-7

**Published:** 2022-12-02

**Authors:** Shahzaib Maqbool, Muhammad Nadeem, Ahmad Shahroz, Kiran Naimat, Imran Khan, Hassaan Tahir, Abdur Rehman, Faiz Anwer, Raheel Iftikhar, Ka Yiu Lee

**Affiliations:** 1 Graduate of Rawalpindi Medical University, RMU, Rawalpindi, Pakistan; 2 Postgraduate Resident Medicine, Bolan Medical Complex, Quetta, Pakistan; 3 Graduate of Liaquat, University of Medical and Health Sciences, Liaquat, Pakistan; 4 Resident Medical Officer, Sandeman Provincial Hospital, Quetta, Pakistan; 5 Department of Hematology and Medical Oncology, Taussig Cancer Institute, Cleveland Clinic, Cleveland, OH USA; 6 Armed Forces Bone Marrow Transplant Centre, Rawalpindi, Pakistan; 7 Department of Health Sciences, Mid Sweden University, Östersund, Sweden

**Keywords:** Autologous stem cell transplantation, Allogeneic stem cell transplantation, Hematopoietic stem cells transplantation, Engraftment syndrome, Acute Graft-vs-Host disease

## Abstract

Engraftment syndrome (ES) is a non-infectious complication seen both in autologous and allogeneic hematopoietic stem cell transplants and is characterized by the presence of non-infectious fever, diarrhea, skin rash, pulmonary infiltration, pulmonary edema, and deranged renal and liver function tests This review will be delineating the incidence of ES, important differential diagnoses to be considered and management options. The literature search was done through various databases like PubMed, Google scholar, Cochrane library, and EMBASE. The incidence of engraftment syndrome was ranging from 8 to 50% in patients undergoing Autologous stem cell transplantation while the incidence was 10–77% in patients undergoing Allogeneic stem cell transplantation. Fever was the most commonly observed symptom of ES in both Autologous and Allogeneic stem cell transplantation while the second most frequently reported symptom was non-infectious diarrhea in patients undergoing autologous stem cell transplantation and Skin rash in patients with Allogeneic stem cell transplantation. Pro-inflammatory cytokines and immune response dysregulation were highlighted as the mechanism behind ES development. The significant difference between ES and aGVHD was observed based on cytokines, with IL-12, IL-1β, IL-6, TNF-α, and IFN-γ levels in plasma being higher in patients with ES as compared to patients with aGVHD. Intravenous methylprednisolone was used as the treatment of choice in the majority of the studies. Overall the incidence of ES was high in patients undergoing allogeneic hematopoietic stem cells transplantation. The survival in patients developing ES was less compared to those who did not develop ES. Engraftment syndrome is one of the complications following hematopoietic stem cell transplantation that need early identification, differentiation from infectious complications, and aGVHD and timely initiation of corticosteroids therapy.

## Introduction

Hematopoietic stem cell transplantation (HSCT) is a medical procedure that is commonly used as an excellent resort for the treatment of various malignancies by infusing stem cells following chemotherapy or radiotherapy [1]. E. Donnall Thomas carried out the first procedure of HSCT in 1957; this was regarded as a revolutionary step toward therapeutic advances in cancer management [2]. According to recent statistics in the year 2019 by World Health Organization (WHO), about 50,000 HSCT procedures are being performed annually [3]. Two types of HSCT have been described, one is autologous stem cell transplantation (Auto-SCT) which uses the recipient stem cells, and the other is allogeneic stem cell transplantation (Allo-SCT) which uses the stem cells from matched or unrelated human leukocyte antigen (HLA) compatible donors [4]. Though HSCT is not only paving the way toward advanced management of various malignant and benign diseases but also posing some significant adverse events and engraftment syndrome is one of the serious side effect profiles associated with HSCT.

Engraftment syndrome is a group of clinical signs and symptoms associated with the process of neutrophil recovery after HSCT [5]. Engraftment syndrome presented with symptoms like non-infectious fever, rash, pulmonary infiltration, or edema that are found to be in close association with HSCT outcome measures [6]. Though some patients developed limited featured ES it has also been associated with transplant-related mortalities [7]. A strong association between ES and acute graft vs host reaction (aGVHD) has been studied, but the cytokine profile is suitable enough to allow the differentiation between these two entities. So, hypothesizing that aGVHD and ES are two different disorders would be of great value to the literature and future perspectives related to ES [8].

We aim this review to summarize the ES incidence, pathogenesis, diagnostic and therapeutic profile of ES, along with aGVHD differentiation from ES in HSCT. This review will also aim to highlight the signs and symptoms variation of ES between Autologous and Allogeneic stem cell transplantation with various therapeutic approaches and benefits in ES.

## Materials and methods

### Search strategies

This study was conducted following the Preferred Reporting Items for Systematic Reviews and Meta-Analyses (PRISMA) guidelines [9]. A comprehensive literature search was done from July 21, 2022, to September 22, 2022. The literature search for this systematic review was done through various databases like PubMed, Google scholar, Web of Science, EMBASE, and Cochrane database by using MeSH key terms of engraftment syndrome (ES), Autologous stem cell transplantation (ASCT), Allogeneic stem cell transplantation, and Hematopoietic stem cell transplantation (HSCT), Graft vs host disease (GVHD). After careful consideration of inclusion and exclusion criteria, a total of 12 studies were included to synthesize this systematic review, and studies involving the occurrence of ES in both Autologous stem cell transplantation or Allogeneic stem cell transplantation were included.

### Inclusion and exclusion criteria

This study will include studies involving patients of both adult and pediatric age groups having different types of hematological and non-hematological malignancies undergoing Autologous stem cell transplantation (Auto-SCT) and Allogeneic stem cell transplantation (Allo-SCT) for any of the disease either hematological malignancies or non-hematological benign and malignant disorders. The studies related to ES from past twenty years were included. The studies in which patients were undergoing conventional chemotherapeutic treatment were excluded and those lacking follow-up after ASCT or allogeneic stem cell transplantation were also excluded.

#### Quality assessment of studies

The quality assessment of involved studies was done by two independent reviewers selected based on competency in the field of research. For quality assessment Newcastle–Ottawa scale (NOS) and Jadad five-item scale was used and studies like RCT, meta-analysis, systematic reviews, case–control, and cohort studies were included while short Communications, letter to the editor, commentaries, unpublished articles, and studies with language other than English were excluded. Studies with a score ≤ 4 (low quality) were excluded while studies with a score ≥ 6 (high quality) were included for the synthesis of this systematic review.

### Data extraction

**Fig. 1:**
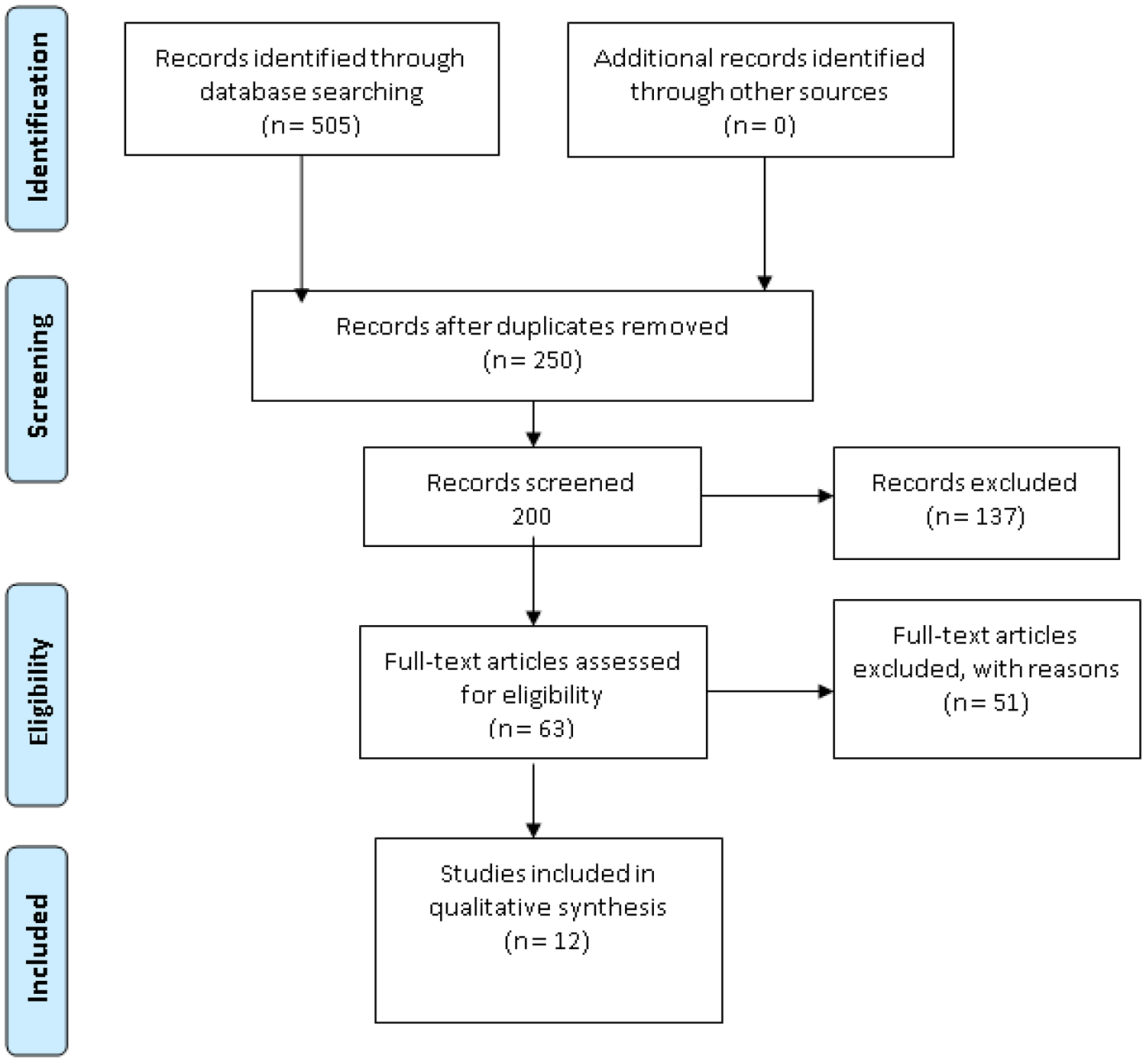
PRISMA flow diagram of studies selection

Data extraction was done independently by two investigators and studies showing the association or occurrence of ES after autologous and Allogeneic stem cell transplantation were selected. The data extraction regarding study name, year of study, type of study, country of origin, ES incidence, ES diagnosis, ES treatment, signs and symptoms of ES, and finally conditioning regimens and aGVHD prophylactic treatment used in Allogeneic stem cell transplantation were extracted and data was arranged in tabulated configuration (Fig. 1).

### Diagnostic criteria leading to engraftment syndrome diagnosis

**Fig. 2:**
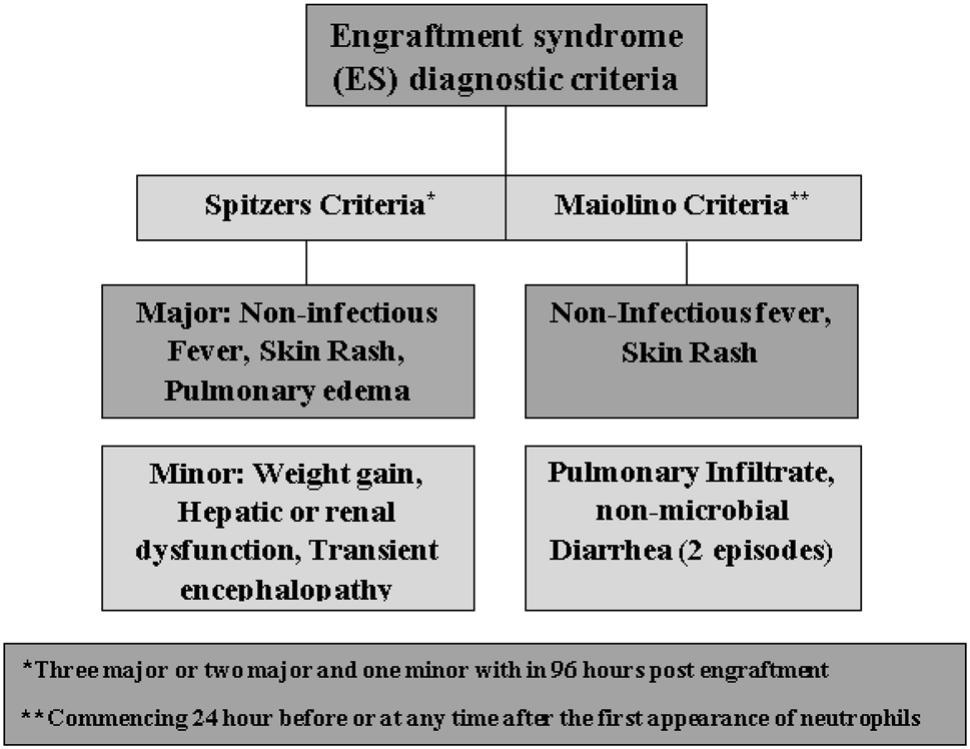
Showing the diagnostic criteria of Engraftment Syndrome after Hematopoietic stem cell transplantation (Auto-SCT, Allo-SCT)

To date, various diagnostic criteria have been proposed to simplify the correct definition of ES, however; Spitzer [5] and Maiolino [10] criteria are the most commonly used criteria to define ES in clinical settings. The presence of non-infectious fever (38 °C), non-infectious diarrhea having 2 or more episodes, and Maculo-papular exanthema rash involving over 25% of body surface area, were the common fractures of ES between Spitzer and Maiolino criteria. However, pulmonary edema of non-cardiogenic origin, weight gain of over 2.5% of the basal level, deranged liver function tests (bilirubin ≥ 2 mg/100 ml and ALT and AST 2 time of the normal), deranged Renal function tests (creatinine two times of the normal value), and Transient encephalopathy of unknown origin was solely described in Spitzer criteria as compared to Maiolino criteria. The flow sheet of Engraftment syndrome criteria is given in Fig. 2 below.

## Results

**Fig. 3:**
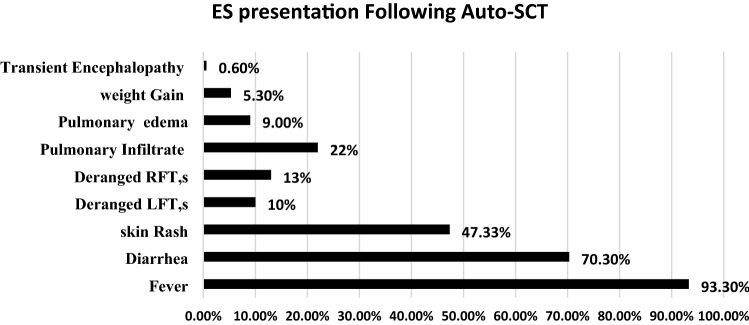
Showing the sign and symptom of Engraftment syndrome following Auto-HSCT

**Fig. 4:**
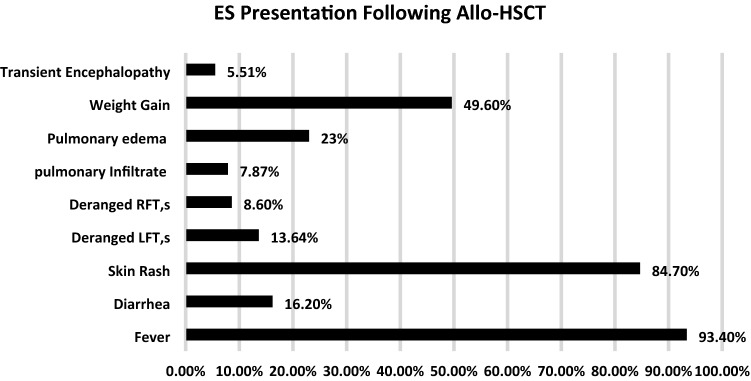
Showing the signs and symptoms of Engraftment syndrome following Auto-HSCT

Engraftment syndrome (ES) is a well-known complication that is found to be associated with Hematopoietic stem cell transplantation (Auto-HSCT and Allo-HSCT) that is manifested through a set of clinically significant signs and symptoms like non-infectious fever, non-infectious diarrhea, skin rash, pulmonary infiltration or edema, weight gain and deranged RFTs, and LFTs [11]. The exact pathophysiological mechanism leading to ES is still unclear however; studies have evaluated the association of various pro-inflammatory cytokines such as IL-2, IL-6, IL-8, INF, and TNF-alpha with the development of ES following hematopoietic stem cell transplantation [12]. ES is also characterized as a constellating set of symptoms occurring during the recovery of neutrophils following Autologous and Allogeneic stem cell transplantation. The commutative incidence of ES following hematopoietic stem cell transplantation has been reported as 5% to 75% as reported in various studies [13–15] (Figs. 3 and 4).

### Engraftment syndrome following autologous and allogeneic stem cell transplantation

See Tables 1, 2, 3, 4, 5.

**Table 1:** Autologous hematopoietic stem cell transplantation indications and engraftment syndrome

Author/year of study	Country	Indications of Auto-SCT	Transplant Type	Total population	Es/no ES
Katzel et al. (2006) [30]	USA	MM	Autologous (Auto-SCT)	90 (100%)	9(10%)/80(90%)
Cornell et al. (2013) [21]	USA	MM/HL/NHL	Autologous (Auto-SCT)	591 (100%)	131(22%)/460(78%)
Gonzalo et al. (2018) [31]	Spain	MM	Autologous (Auto-SCT)	170 (100%)	73 (43%)/97(57%)
Irazabal et al. (2011) [32]	USA	Amyloidosis	Autologous (Auto-SCT)	377 (100%)	29 (8%)/348(92%)
Crreras et al. (2010) [33]	Spain	Amyloidosis, MM, HL, NHL, POEMS, CLL, acute leukemia	Autologous (Auto-SCT)	328 (100%)	42^*^(12.8%)/328 (87.2%)
Dispenzieri et al. (2008) [34]	USA	POEM syndrome	Autologous (Auto-SCT)	30 (100%)	15 (50%)/15 (50%)

MM: Multiple Myeloma, HL: Hodgkin Lymphoma, NHL: Non-Hodgkin Lymphoma, CLL: Chronic Lymphoblastic Leukaemia, POEMS: Polyneuropathy, Organomegaly, Endocrinopathy, Monoclonal protein, Skin changes, ASCT: Autologous Stem Cell Transplantation, ES: Engraftment syndrome. (^*^) represent the exclusion of 1 patient who did not develop fever due to prior 48 h administration of steroids so, a total of 42 patients were included out of 43 (42/43)

**Table 2:** Autologous hematopoietic stem cell transplantation indications and engraftment syndrome incidence, signs and symptoms and response to corticosteroid therapy

Author/year of study	Country	Indications of Auto-SCT	ES signs and symptoms in diseased cohorts	Incidence of ES	Corticosteroid response
Katzel et al. (2006) [30]	USA	MM	Fever (9/9,100%), Diarrhea (8/9, 89%), skin rash (4/9, 44.4%), Pulmonary infiltrated (6/9, 67%)	10%	Responsive
Cornell et al. (2013) [21]	USA	MM/HL/NHL	Fever (118/131, 90%), Diarrhea (89/131, 68%), skin rash (66/131, 50%), abnormal LFT’s (30/131, 23%), Pulmonary infiltrate (20/131, 15%)	22%	Responsive
Gonzalo et al. (2018) [31]	Spain	MM	Fever (73/73, 100%), Diarrhea (66/73, 90%), skin rash (24/73, 33%), Pulmonary infiltrate (24/73, 33%)	43%	Responsive
Irazabal et al. (2011) [32]	USA	Amyloidosis	Fever (24/29, 83%), Diarrhea (20/29, 69%), skin rash (14/29, 48.2%), Pulmonary edema (27/29, 93%), abnormal RFT’s (27/29, 93%)	8%	NA
Crreras et al. (2010) [33]	Spain	Amyloidosis, MM, HL, NHL, POEMS, CLL, acute leukemia	Fever (42/43, 98%), Diarrhea (17/43, 40%), skin rash (28/43, 65%), abnormal LFT’s (9/43, 21%), Pulmonary infiltrate (16/43, 37%), abnormal RFT’s (12/43, 26%), weight gain (8/43, 19%), TA (2/43, 3%)	12.8%	Responsive
Dispenzieri et al. (2008) [34]	USA	POEM syndrome	Fever (14/15, 93%), Diarrhea (11/15, 77%), skin rash (6/15, 43%), weight gain (8/15, 53%)	50%	Responsive

MM: Multiple Myeloma, HL: Hodgkin Lymphoma, NHL: Non-Hodgkin Lymphoma, CLL: Chronic Lymphoblastic Leukaemia, POEMS: Polyneuropathy, Organomegaly, Endocrinopathy, Monoclonal protein, Skin changes, LFT, s: Liver Function Tests, RFT.s: Renal Function Tests

**Table 3:** Allogenic hematopoietic stem cell transplantation (Allo-SCT) indications and engraftment syndrome incidence following Allo-SCT

Author/year of study	Country	Indications of Allo-SCT	Transplant Type	Total population	Es/no ES	Incidence of ES (%)
Lleri et al. (2016) [35]	Turkey	AML, ALL, MDS, Hemoglobinopathies, Aplastic anemia, HLH, Dyskeratosis congenita	Allogenic (Allo-SCT)	169 (100%)	17 (10%)/152 (90%)	10
Omer et al. (2014) [36]	USA	AML, ALL, CML, CLL, MDS, NHL, HL, MM, Amyloidosis, MF	Allogenic (Allo-SCT)	217 (100)	48 (22%)/169 (78%)	22
Chang et al. (2014) [37]	USA	AML, ALL, CML, CLL, MDS, NHL, HL, MM, Histiocytic sarcoma, MF, MPD, and non-malignant diseases^*^	Allogenic (Allo-SCT)	927 (100%)	119 (12.8%)/808 (87.2%)	12.8
Park et al. (2013) [38]	Korea	AML, ALL, CML, MDS, Aplastic anemia, and other diseases	Allogenic (Allo-SCT)	381 (100%)	102 (27%)/279 (73%)	27
Wang et al. (2012) [39]	China	AML, ALL, ABL, CML, MDS, NHL	Allogenic (Allo-SCT)	81 (100%)	51 (63%)/30 (37%)	63
Kanda et al. (2013) [40]	USA	AML, ALL, MLAL, CML, MDS, NHL, malignant lymphoma	Allogenic (All0-SCT)	57 (100%)	44 (77%)/ 57 (23%)	77

MM: Multiple Myeloma, HL: Hodgkin Lymphoma, NHL: Non-Hodgkin Lymphoma, CLL: Chronic Lymphoblastic Leukaemia, CML: Chronic Myeloid Leukaemia, AML: acute myeloid leukemia, ALL: acute lymphoblastic leukemia, MDS: Myelodysplastic syndrome, MF: Myelofibrosis, MPD: Myeloproliferative disorder

**Table 4:** Allogenic hematopoietic stem cell transplantation and conditioning regimen following engraftment syndrome signs and symptoms along with acute graft vs host disease prophylaxis

Author/year of study	Country	ES signs and symptoms in diseased cohorts	Conditioning regimen	aGVHD prophylaxis	Corticosteroid Treatment
Lleri et al. (2016) [35]	Turkey	Fever (17/17, 100%), pulmonary infiltrate (5/17, 29.4%), skin rash (13/17, 76.4%), weight gain (12/17, 70.5%), abnormal RFT, s (6/17, 35.2%), abnormal LFT, s (2/17, 11.7%)	MAC: 82% NMAC: 18%	CsA40.8% CsA + MTX:59.2%	IV Methylprednisolone1-2 mg/kg every 12 h for 7–14 days given in (13/17, 76.5%) of the participants
Omer et al. (2014) [36]	USA	Fever (47/48, 97.9%), pulmonary infiltrate (25/48, 52.1%), skin rash (39/48, 81.3%), weight gain (35/48, 72.9%), abnormal RFT, s (13/48, 27.1%), abnormal LFT, s (10/48, 20.8%), TE (5/48, 10.4%)	MAC: 38.7% NMAC: 61.3%	CsA39.6% CsA + MTX:13.8% CsA + MMF:15.7%	IV Methylprednisolone 1 mg/kg/day for 14 days given in (34/48, 71%) of the participants
Chang et al. (2014) [37]	USA	Fever (119/119, 100%), pulmonary edema (64/119, 54%), skin rash (100/119, 84%), weight gain (91/119, 77%), abnormal RFT, s (9/119, 8%), abnormal LFT, s (29/119, 24%), TE (15/119, 13%)	MAC: 63.1% RIC: 36.9%	Tac + MTX:59.6% Tac + MMF:37.9%	IV Methylprednisolone 1.1 mg/kg/day for 28 days given in (94/119, 79%) of the participants
Park et al. (2013) [38]	Korea	Fever (96/102, 94%), diarrhea (30/102, 29%), pulmonary edema (13/102, 13.3%), skin rash (84/102, 82%), weight gain (28/102, 27.3%)	MAC: 68.5% RIC: 31.5%	MTX STR, NOS	IV Unspecified systemic steroid (1 mg/kg/day) for 7 days given in (74/102, 72.5%) of the participants
Wang et al. (2011) [39]	China	Fever (46/51, 85%), diarrhea (18/51, 35.3%), skin rash (46/51, 85%), weight gain (13/51, 25.5%), abnormal LFT, s (3/51, 6%), TE (1/51, 1.96%)	MAC: 89% RIC: 11%	CsA + MMF:100%	IV Methylprednisolone 1 mg/kg/day for 7 days given in (47/51, 92%) of the participants
Kanda et al. (2013) [40]	USA	Fever (31/44, 70.4%), diarrhea (14/44, 31.8%), skin rash (41/44, 93.1%), weight gain (10/44, 23%), abnormal LFT, s (8/44, 18%), abnormal RFT, s (5/44, 11.36%), PE (11/44, 25%)	MAC: 100% RIC: 0.0%	Tac + MMF:61% CsA + MMF:39%	IV Unspecified systemic steroid 1 mg/kg/day given in (24/44, 54.5%) of the participants

MM: Multiple Myeloma, HL: Hodgkin Lymphoma, NHL: Non-Hodgkin Lymphoma, CLL: Chronic Lymphoblastic Leukaemia, CML: Chronic Myeloid Leukaemia, AML: acute myeloid leukemia, ALL: acute lymphoblastic leukemia, MDS: Myelodysplastic syndrome, MF: Myelofibrosis, MPD: Myeloproliferative disorder, MAC: myeloablative conditioning, RIC: reduced-intensity conditioning, TE: Transient encephalopathy, PE: Pulmonary edema, LFT, s: Liver Function Tests, RFT.s: Renal Function Tests, Tac: Tacrolimus, CsA: Cyclosporine, MMF: mycophenolate mofetil, MTX: methotrexate, NMAC: non-myeloablative conditioning

**Table 5:** Allogenic hematopoietic stem cell transplantation and engraftment syndrome, aGVHD incidence and description of conditioning regimen and aGVHD prophylaxis

Author/year of study	Country	Conditioning regimen	Conditioning regimen detail	aGVHD prophylaxis	aGVHD detail	aGVHD incidence
Ileri et al. (2016) [35]	Turkey	MAC: 82% NMAC: 18%	ATG-based conditioning regimen, BU-based conditioning regimen	CsA40.8% CsA + MTX:59.2%	Cyclosporine alone Cyclosporine: (3 mg/kg/day) on day -1 (PA) + Methotrexate: on day 1,3,6	The cumulative incidence of aGVHD grade II-IV was 12.4% on the 30th-day post-post-transplantation
Omer et al. (2014) [36]	USA	MAC: 38.7% NMAC: 61.3%	**MAC:** TBI-based conditioning regimen + CY combined with high dose BU Or **NMAC:** High dose CY and fludarabine + low dose BU	CsA39.6% CsA + MTX:13.8% CsA + MMF:15.7%	Cyclosporine 2.5 mg/kg/day alone Cyclosporine and methotrexate combined Cyclosporine and mycophenolate mofetil combined	The incidence of aGVHD grade II-IV was 31% in ES ( +) and 23% in non-ES patients at 180th days post-post-transplantation
Chang et al. (2014) [37]	USA	MAC: 63.1% RIC: 36.9%	**MAC:** TBI ≥ 1200 Gy, CY 200 mg/kg, BU 16 mg/kg **RIC:** TBI reduced to 30% of MA and consisted mainly of FU + reduced doses of BU and Thiotepa	Tac + MTX:59.7% Tac + MMF:40.3%	Calcineurin inhibitor plus Methotrexate (n = 71) Calcineurin plus Mycophenolate mofetil (n = 48)	The cumulative incidence of aGVHD grade II-IV was 74% on the 28th-day post-post-transplantation
Park et al. (2013) [38]	Korea	MAC: 68.5% RIC: 31.5%	**MAC:** TBI 13.2 Gy, CY 120 mg/kg, BU 16 mg/kg **RIC:** low dose TBI, BU 9 mg/kg, MEL 150 mg/m^2^	MTX STR	Methotrexate and some non-specified steroids	The cumulative incidence of aGVHD grade II-IV was 56% on the 100th day post-post-transplantation
Wang et al. (2011) [39]	China	MAC: 89% RIC: 11%	**MAC:** BU/CY, BU/CY + ATG, TBI + Ara-c + CY, Flu + BU + CY **RIC:** Flu + BU + TBI + ATG, Flu + BU + CY + TBI	CsA + MMF:100%	IV cyclosporine 3 mg/kg/day at day 1 followed by oral CsA 200-400 ng/ml for 1 month + Mycophenolate mofetil 20-30 mg/kg on day + 1	The cumulative incidence of aGVHD grade II-IV was 35.5% on the 100th day post-post-transplantation
Kanda et al. (2013) [40]	USA	MAC: 100% RIC: 0.0%	**MAC:** FLU/TBI (n = 26), FLU/TBI/CY(n = 16), FLU/TBI/THIO (n = 7), FLU/TBI/MEL (n = 2), TBI/CY/ATG (n = 3), TBI/MEL/ATG (n = 3) **RIC:** Not used in any patient	Tac + MMF:61% CsA + MMF:39%	IV Tacrolimus (n = 35) combined with mycophenolate mofetil and Cyclosporine (n = 22) in combination with mycophenolate mofetil	Incidence of aGVHD grade II-IV was 61% in ES ( +), 36% for III-IV grade aGVHD at 100th-day post-post-transplantation

Tac: Tacrolimus, CsA: Cyclosporine, MMF: mycophenolate mofetil, MTX: methotrexate, NMAC: non-myeloablative conditioning, MAC: myeloablative conditioning, RIC: reduced-intensity conditioning, MEL: melphalan, BU: busulfan, ATG: anti-thymocyte globulin, TBI: total body irradiation, FLU: Fludarabine, STR: steroid

## Discussion

Engraftment syndrome (ES) is a well-known complication that is found to be associated with Hematopoietic stem cell transplantation (Auto-HSCT and Allo-HSCT) that is manifested through a set of clinically significant signs and symptoms. Engraftment syndrome (ES) is a non-infectious complication seen both in autologous and allogeneic hematopoietic stem cell transplants and is characterized by the presence of non-infectious fever, diarrhea, skin rash, pulmonary infiltration, pulmonary edema, and deranged renal and liver function tests.

### Pathophysiological mechanism of engraftment syndrome

Various animal model studies and human model studies have been performed to elaborate on the exact mechanism of ES, still very unclear to label the exact mechanism of ES. However, various human model studies have shown the role of the immune system in the development of ES even in Autologous stem cell transplantation or even in HLA-absent or minor histocompatibility mismatch cases [16]. Various studies have delineated the role of various pro-inflammatory cytokines like IL-1, TNFα, IFN-γ, and IL-12 along with immune system dysregulation [17]. However, studies have also evaluated the role of various other cytokines profiles that were found to be high in isolated ES as compared to aGVHD cytokine profiles including IL-1β, IL-6, IL-12, IL-4, and IL-13 [18]. The presence of higher levels of IL-1β in ES was a leading point toward the association of inflammasome-mediated inflammation and ES development. A proposed hypothetical model involving the exact pathophysiological Mechanism of ES was cytokine-mediated enhanced antigen presentation to T-cells with enhanced T-cells activation and graft rejection in allogeneic settings and reduced tolerance in the autologous setting. With reduced effects of regulatory T-cell (Treg) functions, the T-cells destined to recognize self-MHC and self-peptides become Cytotoxic T-cells with tissue destruction and ultimately graft rejection and ES [16]. There is still more work pending to elaborate the exact mechanism of ES while considering the hypothetical role of various cytokines and immune system dysregulation with reduced Treg functions.

### Engraftment syndrome (ES) vs acute Graft-vs-Host disease (aGVHD) and differentiating role of cytokines

There have been a lot of discussions to differentiate between ES and aGVHD, and various studies have tried to explain this difference and got fruitful results, but how these two terms are different and which pathophysiological Mechanism is involved in both ES and aGVHD it’s still very unclear. However, with the progress in this field, studies have shown the difference in inflammatory and immunological responses between ES and aGVHD. A study by Khandelwal et al., involving the pediatric population has shown the difference of various pro-inflammatory cytokines in the development of isolated ES and isolated aGVHD, which was showing the significant difference of inflammatory response in terms of pro-inflammatory cytokines between aGVHD and isolated ES with higher plasma concentrations of IL-12, IL-1β, IL-6, TNFα, and IFN-γ in patients with ES as compared to aGVHD when measured at day zero to week 8 following Hematopoietic stem cell transplantation [19]. Similarly, a study by Konuma et al. was also showing higher plasma levels of IL-6, IL-12, TNFα, and IFN-γ in patients with engraftment syndrome validating the role of pro-inflammatory cytokines in the development of ES as compared to aGVHD which also shows higher levels of cytokines but not higher than ES [20].

### Management profile of engraftment syndrome (ES) and acute graft-vs-host disease (aGVHD)

The management strategies of ES are mainly based on corticosteroid-based treatment, which is started based on a diagnosis of ES while ruling out the other potential causes of clinical symptomatology. A corticosteroid-based therapy is used widely in the effective treatment of ES either following Autologous hematopoietic stem cell transplantation or Allogeneic Hematopoietic stem cell transplantation. The initiation of methylprednisolone 1–1.5 mg/kg/day until the symptoms are resolving; which typically occurs within 2–3 days, followed by a reduction to 40–50 mg PO Prednisone/day for 2–3 days which typically occurs within 2–3 days is considered a good treatment strategy to mitigate the devastating effects of ES [21]. Similarly, studies have evaluated that early initiation of corticosteroid therapy is associated significantly with a reduction in disease progression and severity. According to Sheth et al., early initiation of methylprednisolone 1 mg/kg/day for 3 days while tapering the dose to 0.5 mg/kg/day over 5–7 days was significantly associated with a reduction in ES-related complications and early recovery [22].

In the same vein, the association of ES with aGVHD in patients undergoing Allogeneic Hematopoietic stem cell transplantation is also a factor leading to post-transplantation complications. The utilization of aGVHD prophylaxis is of paramount significance to prevent transplant-related rejections and mortalities. While knowing the role of immune response dysregulation involving effector T-cells, various prophylactic treatment options have revolutionized the prevention of ES/aGVHD [23]. Using T-cell suppression effect through Tacrolimus (Tac) and Cyclosporine (Cys) in combination with methotrexate (MTX) and mycophenolate mofetil (MMF) are the best-known regimens used to prevent aGVHD. According to two RCTs conducted in 1990, the combination of Tac/MTX was the most effective combination used for the treatment of grade II and grade III GVHD as compared to Cys/MTX combination [24, 25].

There are various other treatment strategies now become available for the effective management of GVHD grade II–IV. New advances in aGVHD include the utilization of post-transplant cyclophosphamide at doses of 50 mg/kg on days + 3 and + 4 following the infusion of haploidentical stem cells was associated with a reduction in aGVHD [26]. Similarly, anti-thymocyte globulin (ATG) [27], sirolimus (a mTOR inhibitor) [28], along with select and pan T-cells depletion strategies are now proving fruitful, however; using Tac/MTX/MFM and Cys/MTX/MFM are still considered standard regimens in the prophylaxis of aGVHD [29]. All these strategies help in preventing the hyperactivity of the innate immune system with a significant reduction in cases of ES and aGVHD with improved overall survival (OS) following hematopoietic stem cell transplantation.

## Conclusion

Engraftment Syndrome and acute Graft-vs-Host disease are the commonly encountered complications after hematopoietic stem cell transplantation. The mechanism involving these complications is hyperactivity of the innate immune system and pro-inflammatory cytokines storm that predisposed the patients to develop ES/aGVHD following Hematopoietic stem cell transplantation. In this review synthesis, the most common presentation of ES was non-infectious fever, diarrhea, and skin rash following Autologous hematopoietic stem cell transplantation non-infectious fever, and skin rash followed by weight gain was most commonly observed ES presentation after Allogeneic stem cell transplantation. Variable values of ES incidence were observed in this review ranging from 8 to 77% while using the Spitzer and Maiolino criteria of ES. The therapeutic use of corticosteroids, particularly intravenous methylprednisolone at a higher starting dose and followed by tapering, was highly effective in combating ES. Similarly, the use of immunosuppressive therapy was also highly effective in combating aGVHD. The difference between ES and aGVHD was evaluated based on plasma concentrations of various pro-inflammatory cytokines, which were present in higher concentrations in patients with ES as compared to aGVHD patients.

## Data Availability

All the data generated or analyzed during this study are included in this manuscript.

## Abbreviations

MM: Multiple Myeloma
HL: Hodgkin Lymphoma
NHL: Non-Hodgkin Lymphoma
CLL: Chronic Lymphoblastic Leukaemia
POEMS: Polyneuropathy, Organomegaly, Endocrinopathy, Monoclonal protein, Skin changes
ASCT: Autologous Stem Cell Transplantation
ES: Engraftment syndrome
CML: Chronic Myeloid Leukaemia
AML: Acute myeloid leukemia
ALL: Acute lymphoblastic leukemia
MDS: Myelodysplastic syndrome
MF: Myelofibrosis
MPD: Myeloproliferative disorder
MAC: Myeloablative conditioning
RIC: Reduced-intensity conditioning
TE: Transient encephalopathy
PE: Pulmonary edema
LFTs: Liver Function Tests
RFTs: Renal Function Tests
Tac: Tacrolimus
CsA: Cyclosporine
MMF: Mycophenolate mofetil
MTX: Methotrexate
NMAC: Non-myeloablative conditioning
MEL: Melphalan
BU: Busulfan
ATG: Anti-thymocyte globulin
TBI: Total body irradiation
FLU: Fludarabine
STR: Steroid
